# Radiographic and clinical predictors of surgical outcomes following endoscopic decompression for radiculopathy in adult degenerative scoliosis: A multi-center retrospective study

**DOI:** 10.1016/j.bas.2026.105990

**Published:** 2026-03-03

**Authors:** Mounica Paturu, Joshua Woo, Kosuke Saguira, David Huie, Christoph Hofstetter, Muhammad M. Abd-el-Barr

**Affiliations:** a Department of Neurosurgery, Duke University Medical Center, Durham, NC, USA; b Department of Neurosurgery, University of Washington, Seattle, WA, USA

**Keywords:** Endoscopic decompression, Adult degenerative scoliosis, Radiculopathy, Transforaminal decompression, Minimally invasive spine surgery, Foraminal stenosis

## Abstract

**Introduction:**

Endoscopic transforaminal decompression is a minimally invasive approach for treating radiculopathy in adult degenerative scoliosis (ADS) secondary to foraminal stenosis. Previous research supports the feasibility and safety of this technique, but the correlation of preoperative measures with postoperative outcomes remains unclear.

**Research question:**

This study aims to assess the efficacy of endoscopic decompression for ADS-related radiculopathy and identify predictive factors for postoperative pain and functionality to optimize surgical decision-making.

**Materials and methods:**

ADS patients with foraminal stenosis and radiculopathy undergoing endoscopic decompression with >6 month follow-up were retrospectively reviewed. Pre-operative spinopelvic measurements, demographics, and symptom predominance were collected and compared to post-operative clinical outcomes (Visual Analog Scale (VAS) and Oswestry Disability Index (ODI) scores).

**Results:**

28 patients underwent endoscopic transforaminal decompression for primary radiculopathy or axial back pain. Mean preoperative ODI was 33 and VAS was 4.7 (back pain) and 6.1 (leg pain). Postoperatively, both VAS scores demonstrated significant improvement. Correlative analysis revealed that increasing deformity severity, measured by Cobb angle, pelvic incidence (PI) and central sacral cervical line (CSVL), was associated with poorer outcomes. Cobb angles from 18° to 25° and 26°- 52° were linked to worse postoperative pain and functional scores. Hyperlordosis (LL > 32°) similarly correlated with inferior outcomes. Conversely, PI values near physiologic range (47°-65°) were associated with greater postoperative functionality.

**Discussion and conclusion:**

This study supports transforaminal endoscopic decompression as an effective treatment for radiculopathy and back pain in ADS. Lower preoperative Cobb angles and mild to moderate spinopelvic alignment yielded better postoperative outcomes. These findings may help guide surgical planning and predict success following endoscopic decompression.

## Highlights

• Transforaminal endoscopic decompression improves pain and function in ADS patients.
• Favorable outcomes are present in patients with Cobb <26°, LL > 32°, PI 47°–65°.
• Leg-dominant pain predicts greater postoperative relief.
• Severe deformity/instability reduces decompression success.
• Radiographic and clinical thresholds can guide patient selection and planning.

## 1 Introduction

ADS is a progressive spinal disorder with a growing prevalence in the aging population, affecting up to 32% of individuals over 65 years (Schwab et al., 2005; Robin et al., 1982; Aebi, 2005). Radiographically defined by a coronal Cobb angle of ≥10° in skeletally mature individuals with no prior history of scoliosis, ADS arises from age-related asymmetric degeneration of intervertebral discs, facet arthropathy, and deterioration of ligamentous and muscular support of the spinal motion segments. These cumulative degenerative changes compromise spinal alignment and stability, resulting in both coronal and sagittal imbalance. The ensuing biomechanical dysfunction frequently contributes to a spectrum of clinical symptoms including axial back pain and radiculopathy, primarily due to neural element compression and dynamic instability across deformed segments (Phillips et al., 2013; Ploumis et al., 2007).

Neurological symptoms in ADS are commonly multifactorial and exacerbated by segmental stenosis, with foraminal narrowing playing a particularly critical role (Buttermann and Mullin, 2008). Foraminal stenosis often occurs on the concave side of the scoliotic curve, where intervertebral disc height collapse, facet joint hypertrophy, osteophyte formation, and vertebral translation contribute to nerve root impingement. Conservative management—including physical therapy, NSAIDs, bracing, and epidural steroid injections—offers limited long-term efficacy in patients with progressive deformity and symptomatic stenosis. As symptoms become refractory, surgical intervention is considered. Traditional approaches—ranging from decompression alone to decompression with instrumented fusion—aim to alleviate neural compression and, when necessary, stabilize or correct spinal malalignment (Shamji et al., 2015; Simotas et al., 2000; Koerner et al., 2015). However, in older adults, such procedures are associated with substantial perioperative morbidity, including significant blood loss, wound complications, prolonged hospitalization, and delayed functional recovery (Wang et al., 2003; Benz et al., 2001).

To mitigate these risks, there has been a paradigm shift toward minimally invasive spine surgery (MISS), which emphasizes reduced iatrogenic tissue disruption while preserving spinal stability. MISS techniques such as minimally invasive transforaminal lumbar interbody fusion (MIS-TLIF), unilateral laminotomy for bilateral decompression, and full-endoscopic transforaminal decompression have been increasingly utilized in the management of lumbar degenerative disease. These approaches have demonstrated favorable perioperative outcomes, including reduced operative time, lower estimated blood loss, shorter hospital stay, and faster postoperative rehabilitation (Fontes and Fessler, 2017; Kato et al., 2017; Kelleher et al., 2010). In the context of ADS, early evidence suggests that percutaneous endoscopic decompression may serve as an effective and less morbid alternative to traditional open procedures for select patients (Hasan et al., 2019).

Several studies have highlighted the clinical utility of endoscopic decompression in ADS. Hasan et al. reported significant improvements in ODI and VAS scores in patients with lumbar scoliosis and radiculopathy following endoscopic intervention (Hasan et al., 2019). Similarly, Ruetten et al. and Zhang et al. demonstrated comparable outcomes between endoscopic decompression and short-segment fusion in stable deformities, with added benefits in complication profile and hospital resource utilization (Ruetten et al., 2009; Zhang et al., 2025). Jin et al. further demonstrated that endoscopic transforaminal decompression provides meaningful symptom relief in scoliotic patients with mild deformities (Jin et al., 2022). Biomechanical analysis has corroborated these findings, suggesting that endoscopic approaches, by preserving posterior tension bands and facet integrity, result in less iatrogenic destabilization relative to open decompression (Farshad et al., 2022). Nevertheless, patient selection remains a significant challenge.

While many studies address fusion vs decompression**,** few have examined endoscopic decompression specifically in ADS**,** and even fewer provide threshold-based guidelines**.** This study begins to fill that gap by offering evidence-based parameters that can enhance personalized surgical planning**.** Furthermore, there is no standardized algorithm or predictive model to determine which patients with ADS are optimal candidates for isolated endoscopic decompression, and to our knowledge, there are no studies that comprehensively evaluate how preoperative spinopelvic parameters and clinical characteristics may affect surgical outcomes. Thus the aim of this multi-surgeon, multi-institutional study is to (1) evaluate the clinical efficacy of transforaminal endoscopic decompression in ADS patients presenting with radiculopathy and/or axial back pain, and (2) identify key radiographic and symptom-based predictors of postoperative improvement. By delineating threshold values for relevant spinopelvic alignment metrics, we aim to establish evidence-based criteria for patient stratification to ultimately promote a data-driven, anatomy-informed approach to surgical decision-making that broadens the applicability of endoscopic decompression in appropriately selected ADS patients.

## 2 Materials and methods

**Fig. 1:**
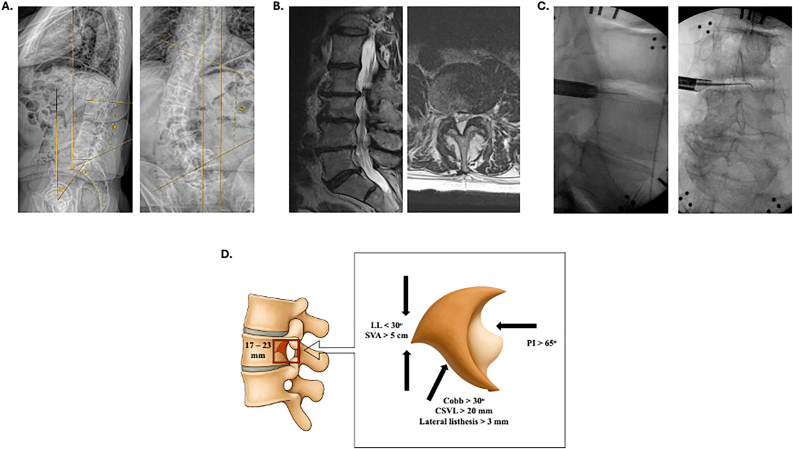
Pre-operative and intra-operative images with spinopelvic parameters. **A)** Full length pre-operative patient sagittal and coronal X-rays of a 68 YOM undergoing L3/4 transforaminal endoscopic decompression. Captured measurements include pelvic incidence (PI), lumbar lordosis (LL), pelvic tilt (PT), sagittal vertical axis (SVA), central sacral vertical line (CSVL), and coronal Cobb angle. **B)** T2 MRI sagittal and axial views of L3/4 herniated disc. **C)** Intra-operative sagittal and coronal fluoroscopy views of localization and discectomy. **D)** Schematic of the lumbar neuroforaminal space and distortion from LL, SVA, coronal Cobb angle, CSVL, lateral listhesis, and PI radiographic measurements.

We performed a multi-center retrospective review of patients (age 18-90 years) in the last 10 years with back and/or radicular pain from lateral recess, foraminal, or extraforaminal stenosis and coexisting ADS (coronal cobb angle >10°) undergoing endoscopic transforaminal decompression. Data from two surgeons at different academic institutions (n = 18 and n = 10, between institutions) were included and patients with prior fusions, infection, tumor, or metastatic disease were excluded. Patient demographics, re-operation status, surgical approach (concave, convex, main/fractional curve), clinical outcomes (VAS and ODI scores), and spinal measurements (pelvic incidence, pelvic tilt, PI/LL mismatch, lumbar lordosis, central sacral vertical line, sagittal vertical line, coronal cobb angle, presence of listhesis) were recorded from pre-operative, initial post-operative, and final post-operative visits (Fig. 1A). Surgical pathology from MRI, CT, and full-length radiographs and spinal parameter measurements were verified by 3 independent neurosurgeons blinded to outcomes (Fig. 1B). Statistical analysis was performed via SPSS.

### 2.1 Surgical indication

All included patients were extensively worked up and only included for endoscopic decompression if pain and symptoms were resistant to conservative measures. Specifically, lower extremity radiculopathy demonstrating no response to prior steroid injections, with corresponding EMG findings and clinical correlate (i.e., via physical exam) was the primary indication for surgery. Indications for operative decompression was driven by clinical presentation and confirmed with pre-operative imaging. The specific level operated on was also confirmed by pre-operative imaging (MRI, and CT SPECT).

### 2.2 Surgical technique

After informed consent was obtained, the patient was brought to the operating room and placed in the prone position on a radiolucent table. All pressure points were padded appropriately. General anesthesia was induced without complications. The back was prepped and draped in the usual sterile fashion.

Using fluoroscopic guidance, the appropriate lumbar disc space was identified and marked. A local anesthetic was infiltrated at the planned entry point approximately 8–10 cm off midline. A spinal needle was inserted and directed toward the Kambin's prism of the designated disc space (Fanous et al., 2019). Once appropriate trajectory and position were confirmed with biplanar fluoroscopy, a guidewire was passed, followed by sequential dilators. In some cases, crown reamers were utilized to widen the Kambin's prism (foraminotomy) by performing a partial resection of the ventrolateral superior articulating facet (SAP). The working channel cannula was then introduced over the dilator and docked onto the SAP of the caudal index level vertebral body (Fig. 1C).

The endoscope system was introduced through the working channel, and continuous irrigation was established. Under direct visualization, soft tissue was cleared to expose the bony anatomy. A high-speed burr was used to complete the foraminotomy, resecting the ventrolateral portion of the SAP and hypertrophic ligamentum flavum. Disc protrusion contributing to foraminal compromise was carefully debulked using pituitary rongeurs and graspers. The surgical endpoint was not objectively defined due to individual case complexity. However, our approach was generally consistent with the literature for endoscopic foraminotomy, which presents the theoretical endpoint as free mobilization of the exiting nerve root, which may be intraoperatively verified by its free movement and strong pulsation (Ahn et al., 2014, 2022, 2025).

Hemostasis was ensured throughout using bipolar cautery and irrigation. The endoscope and cannula were withdrawn. The skin was closed, and a sterile dressing was applied.

## 3 Results

### 3.1 Demographics and surgical data

A total of 28 patients underwent endoscopic decompression, with a nearly equal gender distribution (13 males, 15 females) and a mean age of 67 years (±9.5). The most frequently operated spinal level was L4/5 (n = 13), followed by L5/S1 (n = 8), L2/3 (n = 7), and L3/4 (n = 4). Four patients underwent multilevel decompression procedures, all of which involved two levels. Regarding symptom presentation, most patients reported leg-dominant pain (n = 18, 64%), with fewer experiencing either back-dominant (n = 3, 11%) or equally distributed leg and back pain (n = 7, 25%). Initial postoperative follow-up occurred at an average of 53 days (±48), and the mean duration of final follow-up was 244 days (±171), allowing for assessment of both early and sustained clinical outcomes (Table 1). Average spinopelvic parameters are listed in Table 1 with 36% of patients having lateral listhesis or 39% having spondylolisthesis at the level of surgery.

**Table 1:** Patient demographic and preoperative radiologic characteristics.

Parameter	Value
Total number of patients	28
Gender (Male/Female)	13/15
Mean age (years)	67 ± 9.5
Surgical level (n)	
L2/3	7
L3/4	4
L4/5	13
L5/S1	8
Multi-level decompression	4
Primary pain type	18 Leg/3 Back/7 Equal
Initial follow-up (days)	53 ± 48
Final follow-up (days)	244 ± 171
Radiographic Parameter	
Cobb angle (°)	22.7 ± 11.2
Lumbar lordosis (°)	40 ± 17.1
Pelvic incidence (°)	56 ± 10.4
PI–LL mismatch (°)	18.8 ± 12.3
Pelvic tilt (°)	26.7 ± 6.7
Sagittal vertical axis (cm)	6.16 ± 7.5
Lateral listhesis	Present in 36%
Spondylolisthesis	Present in 39%
Concave (main/fractional)	12/12
Convex (main/fractional)	1/3

Data presented as mean ± standard deviation, unless noted otherwise.

### 3.2 Clinical outcomes

**Fig. 2:**
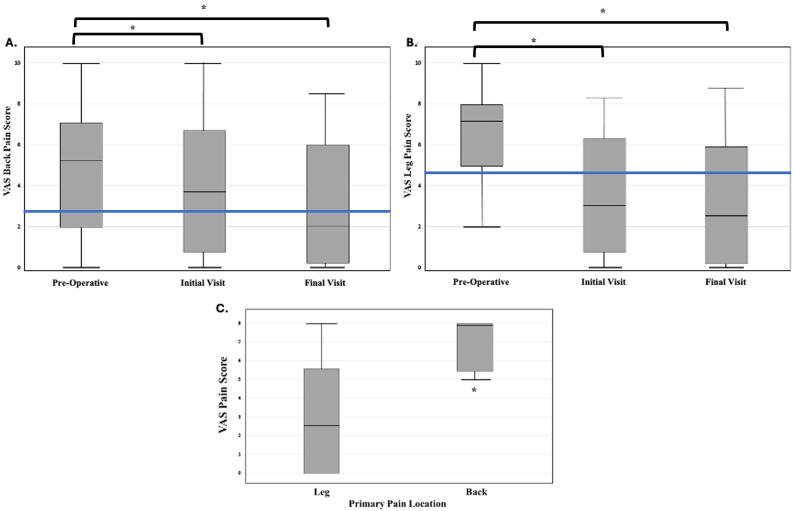
Visual Analogue Score (VAS) Leg and Back Pain scores over time. Box plots depict **A)** VAS-Back Pain and **B)** VAS-Leg Pain at the pre-operative, initial post-operative, and final post-operative visit time points. **C)** Primary pain distribution affecting post-operative Visual Analogue Scores (VAS), box plots depict VAS pain score averages at initial post-operative visit, stratified by reported pre-operative primary pain location (VAS-Leg vs. VAS-Back). * demonstrates statistical significance at p < 0.05.

No post-operative complications were reported, including but not limited to infection, hematoma, CSF leaks, and nerve injury. As shown in Fig. 2, average VAS scores for both back (Fig. 2A) and leg pain (Fig. 2B) significantly decreased from preoperative levels to both initial and final postoperative follow-ups. Mean VAS back pain scores declined from 4.76 ± 3.05 preoperatively to 3.95 ± 3.03 at the initial post-operative visit and 3.14 ± 3.12 at final follow-up (p = 0.048). Similarly, mean VAS leg pain scores showed a substantial reduction from 6.09 ± 2.59 to 3.64 ± 3.01 initially and further down to 3.40 ± 3.31 (p = 0.0009).

ODI scores also trended towards improvement, decreasing from 33.0 ± 14.3 preoperatively to 25.8 ± 13.8 initially and 24.8 ± 18.7 at final follow-up (Table 2). At the final postoperative assessment, 75% of patients achieved the MCID for leg pain and 61% achieved MCID for ODI, reflecting meaningful reduction in both pain and functional status after endoscopic decompression. Fewer (50%) patients reported achieving MCID in back pain reduction.

**Table 2:** Clinical outcomes by pre-operative and post-operative time point.

Outcome Metric	Pre-operative	Initial Follow-up	Final Follow-up
ODI (mean ± SD)	33.0 ± 14.3	25.8 ± 13.8	24.8 ± 18.7
VAS back pain (mean ± SD)	4.76 ± 3.05	3.95 ± 3.03	3.14 ± 3.12
VAS leg pain (mean ± SD)	6.09 ± 2.59	3.64 ± 3.01	3.40 ± 3.31
ODI MCID achieved (%)	–	–	61
VAS leg MCID achieved (%)	–	–	75
VAS back MCID achieved (%)	–	–	50

Data presented as mean ± standard deviation, unless noted otherwise.

ODI = Oswestry Disability Index; VAS = Visual Analogue Score; ODI MCID = ODI minimal clinically important difference; VAS MCID = VAS minimal clinically important difference.

### 3.3 Pain distribution and outcomes

Among patients stratified by primary pain location, postoperative outcomes varied notably between those presenting with leg versus back pain. As shown in Fig. 2C, individuals with primary leg pain demonstrated a broader range of postoperative VAS scores, with a lower median, indicating overall better pain relief at the initial post operative visit (p = 0.041). In contrast, those with primary back pain exhibited consistently higher postoperative VAS scores, with minimal variability. Correspondingly, 75% of patients with leg pain reported improvement, compared to 61% in the back pain cohort. Rates of worsened or unchanged symptoms were also lower in the leg pain group (21% worsened, 3.5% unchanged) versus the back pain group (32% worsened, 7% unchanged) (p = 0.0371) (Table S1).

### 3.4 Radiological predictors and correlations

At final follow-up, correlation analysis revealed statistically significant associations between postoperative pain scores and spinopelvic parameters. Change in back pain demonstrated a moderate negative correlation with PI (r = −0.403, p = 0.0371). Final back pain scores were strongly positively correlated with Cobb angle (r = 0.617, p = 0.0005), and final leg pain scores also showed a strong positive correlation with Cobb angle (r = 0.557, p = 0.0021). Change in leg pain was moderately to strongly correlated with Cobb angle (r = 0.494, p = 0.0076) and strongly correlated with CSVL deviation (r = 0.566, p = 0.0116) (Table 3).

**Table 3:** Significant correlations between pain scores and spinopelvic parameters at last follow-up.

Pain Parameter	Pelvic Parameter	Test Used	Correlation (r)	p-value	Strength and Direction
Back pain Change	PI	Pearson	−0.403	**0.0371**	Moderate Negative
Back Pain	Cobb	Spearman	0.617	**0.0005**	Strong Positive
Leg Pain	Cobb	Spearman	0.557	**0.0021**	Strong Positive
Leg Pain Change Leg Pain Change	Cobb CSVL	Spearman Spearman	0.494 0.566	**0.0076** **0.0116**	Moderate - Strong Positive Strong Positive

*PI = Pelvic incidence; Cobb = Coronal cobb angle; CSVL = central sacral vertical line.

**Fig. 3:**
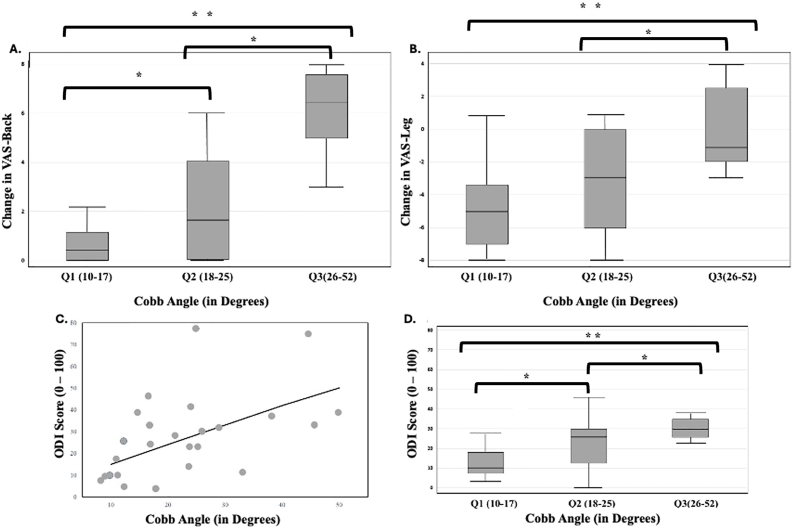
Correlation between pre-operative Coronal Cobb Angle (CCA) and change in VAS pain scores (pre-operative VAS scores subtracted from last post-operative visit VAS scores) for **A)** VAS-Back and **B)** VAS-Leg; **C)** Scatter plot depicting significant correlation between pre-operative CCA and reported ODI scores at the last post-operative visit; **D)** Box plots depicting significant differences in ODI scores and average pre-operative CCA. CCA on full-length x-rays are separated into three bins: Q1-Q3, ranging from 10 to 52°. * demonstrates statistical significance at p < 0.05. **p denotes <0.01.

The mean preoperative Cobb angle among patients was 22.7° ± 11.2°. To further identify threshold correlations between pre-operative spinopelvic parameters and post-operative outcomes, patients were stratified by coronal Cobb angle into three equal quartiles: Q1 (10–17°), Q2 (18–25°), and Q3 (26–52°). Changes in back and leg pain postoperatively demonstrated a significant Cobb angle–dependent relationship. As shown in Fig. 3A, patients in Q1 and Q2 exhibited greater reductions in back pain (p = 0.0005) compared to Q3, where reductions in pain scores were less pronounced. A similar pattern was observed for leg pain (Fig. 3B), with patients in Q3 experiencing the least improvement, with notable increase in overall average pain scores (p = 0.0021). Additionally, ODI scores correlated positively with increasing Cobb angles (p = 0.0039), suggesting worse functional impairment with more severe deformity (Fig. 3C). Boxplot analysis confirmed that patients in Q3 had significantly higher ODI scores than those in Q1 and Q2 (0.0076) (Fig. 3D).

**Fig. 4:**
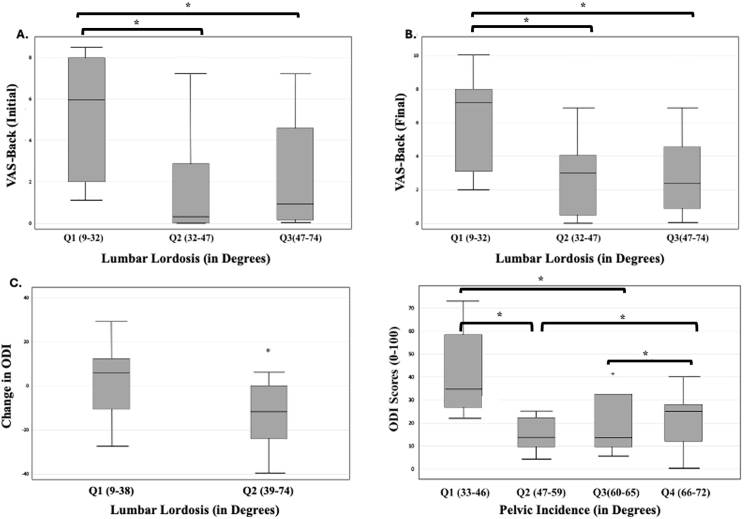
Correlation between pre-operative lumbar lordosis (LL) and **A)** initial post-operative VAS-Back pain score; **B)** final post-operative VAS-Back pain score; and **C)** Change in Oswestry Disability Index (ODI) scores (pre-operative ODI scores subtracted from last post-operative ODI scores). **D)** Correlation between pre-operative pelvic incidence (PI) and functional status, demonstrated by final postoperative ODI score. LL on full-length x-rays are separated into three bins for **(A)** and **(B)** (Q1-Q3, ranging from 9 to 74°) and two bins for **(C)** (Q1-Q2, ranging from 9 to 74°); PI on full-length x-rays are separated into four bins for **(D)**: Q1-Q4, ranging from 33 to 72°. * demonstrates statistical significance at p < 0.05.

LL averaged 40° ± 17.1° preoperatively and postoperative clinical outcomes were analyzed across equal quartiles of LL. Patients with reduced lordosis, particularly those with LL less than 32°, exhibited significantly higher back pain scores at initial (p = 0.0371) and last follow ups (p = 0.0485). Conversely, patients with normal or hyperlordotic alignment (LL 32°–74°) experienced more favorable postoperative back pain scores, as depicted in Fig. 4. Furthermore, those with LL < 38° demonstrated less improvement in ODI at initial follow-up (p = 0.0441). Pelvic incidence was recorded at an average of 56° ± 10.4°, with most patients falling within a range of 33° to 72°. Functional improvement was greatest in those with PI values between 47° and 65°, a range generally considered physiologic (p = 0.0439) (Fig. 4D).

### 3.5 Reoperation profile

**Fig. 5:**
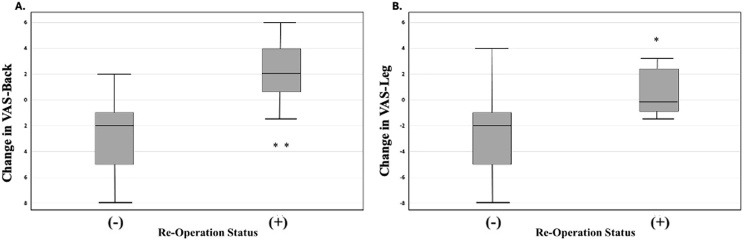
VAS pain scores at last post-operative visit by re-operation status for **A)** VAS-Back and **B)** VAS-Leg. * demonstrates statistical significance at p < 0.05. **p denotes <0.01.

Reoperation was required in 7 cases (25%) and postoperative pain improvement, as measured by changes in VAS back and leg pain scores, was stratified by reoperation status. The average time to re-operation was 272.7 days (Range: 0 – 663 days; Median: 191 days). Table S3 provides a comprehensive overview of the original and revision surgeries. Patients who did not require reoperation (−) demonstrated significantly greater reductions in both back (p = 0.0004) and leg pain (p = 0.0195) compared to those who underwent reoperation (+) (Fig. 5). There was an average VAS back pain increase of 5.1 and leg pain increase of 3.36 between the re-operation vs non re-operation cohorts with the reoperation group exhibiting a broader range of scores, including cases with minimal or negative change in pain scores. These findings suggest that patients who required reoperation experienced less overall improvement in both back and leg pain following their initial procedure.

## 4 Discussion

This study evaluated the clinical efficacy of endoscopic transforaminal decompression in ADS focusing on how specific radiographic and clinical parameters predict postoperative outcomes. Specifically, patients with mild to moderate coronal deformities (Cobb angle <26°), preserved lumbar lordosis (LL > 32°), and normative spinopelvic parameters—particularly pelvic incidence (PI) between 47° and 65°—were significantly more likely to achieve meaningful pain relief and functional improvement following decompression alone. Conversely, patients with substantial deformity or instability, including high Cobb angles, CSVL, lateral listhesis, and spondylolisthesis (Fig. S2), demonstrated limited symptomatic improvement, supporting the hypothesis that increasing structural deformity may limit the clinical utility of decompression. These thresholds help identify which patients may benefit from decompression alone and which may require fusion or more complex realignment procedures, particularly in borderline cases. This study, therefore, provides an important step toward evidence-based guidelines in MIS planning for ADS by translating radiographic and clinical patterns into relevant predictors that can guide treatment algorithms and improve both surgical safety and efficacy.

Spinopelvic alignment is a well-established predictor of surgical outcomes in fusion-based treatment of ADS with PI–LL mismatch >10°, SVA >5 cm, and elevated pelvic tilt associated with poorer function (Schwab et al., 2012; Lafage et al., 2009). However, its predictive value in minimally invasive endoscopic decompression remains less clearly defined. Foundational studies have established the link between scoliosis, foraminal stenosis, and radiculopathy, laying the basis for decompression-based strategies (Pritchett and Bortel, 1993; Ploumis et al., 2011). More recent evidence suggests suboptimal outcomes in patients with Cobb angles >20°, particularly those with coronal imbalance or lateral listhesis (Asada et al., 2024; Dangelmajer et al., 2014). Improved results have been reported when decompression is paired with limited fusion in patients with sagittal malalignment (Asada et al., 2024; Echt et al., 2023; Masuda et al., 2018). Hasan et al. demonstrated that even technically successful decompression yields poor outcomes in patients with instability or misalignment—particularly high PI–LL mismatch and lateral listhesis (Hasan et al., 2019). Park et al. showed that Cobb progression, especially with asymmetric muscle degeneration, reduces symptom durability (Park et al., 2011). Similarly, Hauser et al. found that dynamic instability, including undiagnosed spondylolisthesis or facet joint laxity, increases the risk of recurrent mechanical pain (Hauser et al., 2022). Although decompression may offer minor sagittal realignment in select patients, these changes are limited in advanced deformity (Minamide et al., 2017; Bouknaitir et al., 2022). Modest improvements in global alignment and delayed MCID achievement in patients with poor muscle health further highlight the importance of assessing pre-operative radiographic parameters (Fujii et al., 2015).

Although prior work has identified general associations between deformity and surgical outcomes, this study goes further by quantifying these relationships and linking them to clinical benchmarks, providing specific radiographic thresholds that correlate with a higher likelihood of achieving improved pain and functional outcomes. We also demonstrate, for the first time to our knowledge, a dose-response relationship between increasing deformity and diminished symptomatic and functional improvement, stratified across deformity quartiles. In doing so, these findings not only validate prior biomechanical hypotheses but also offer actionable parameters to support individualized surgical decision-making in minimally invasive treatment of ADS.

Per the Scoliosis Research Society (SRS)-Schwab classification, our patient cohort included mostly patients with L coronal curve type, + PI/LL, +SVA, +PT indicative of moderate deformity and correlated with poor health related quality of life outcomes without intervention and reduced functional status beyond critical thresholds of 22, 47, and 11, respectively, for PT, SVA, and PI/LL (Park et al., 2024). In this cohort, abnormal sagittal alignment displaces the body's center of gravity anteriorly, triggering compensatory muscle activation that leads to chronic fatigue and increased axial loading. Over time, this maladaptive compensation accelerates facet joint degeneration and intervertebral disc collapse, contributing to foraminal and central canal stenosis—both of which are central to the pathogenesis of radicular symptoms in ADS. Consistent with this mechanism, patients in our cohort reported clinically significant radiculopathy, which served as the primary surgical indication.

Given this anatomy and symptomatic profile, all included patients across both institutions in our cohort demonstrated foraminal stenosis, with a subset exhibiting additional recess and/or extraforaminal stenosis (stratified further in our single institution data for n = 18) driving their ADS-associated radiculopathy. Accordingly, a transforaminal approach was employed in all cases, as prior literature supports improved outcomes and post-operative prognosis for this pattern of compression compared to an interlaminar technique (Yin et al., 2020; Takebayashi et al., 2023).

In parallel with the radiographic findings, 65% of our patients reported leg-dominant pain with secondary back pain, while the remainder experienced primarily axial back pain. This distribution aligns with known symptomatology in ADS and can directly be addressed by transforaminal endoscopic decompression. In contrast, back-predominant pain is typically multifactorial, arising from facet arthropathy, muscle atrophy, and spinopelvic misalignment (Sun et al., 2019). Patients with leg-predominant symptoms experienced significantly better postoperative improvement, consistent with prior studies showing that radicular symptoms respond more favorably to decompression than axial pain (Ruetten et al., 2009; Minamide et al., 2017; Qu et al., 2023). Additionally, prior research has shown higher MCID achievement in leg-dominant patients following decompression, underscoring the prognostic value of symptom type (Massel et al., 2020). However, in a subset of patients, decompression provided the secondary benefit of reducing concomitant axial back pain. This improvement likely reflects treatment of a shared pathoanatomic driver, as neural compression can serve as a primary pain generator, and its relief reduces mechanical irritation and local inflammatory signaling. Additionally, symptom overlap and misattribution between radicular and axial pain may contribute, such that surgical resolution of the primary compressive pathology decreases perceived back pain. Decompression may also indirectly reduce nerve-mediated paraspinal muscle guarding, improve segmental biomechanics, and attenuate central sensitization by eliminating the nociceptive source, thereby improving both leg-dominant and axial symptoms. Importantly, although decompression is not indicated for isolated axial low back pain, multiple studies have demonstrated clinically meaningful improvement in concomitant back pain when radiculopathy is the primary pathology (Knight et al., 2023; Wai et al., 2009; Rampersaud et al., 2013). Ultimately, our results reinforce that leg-dominant pain is associated with more discrete, surgically addressable pathology, leading to more reliable and sustained symptom resolution following endoscopic intervention.

The clinical outcomes secondary to radiological findings can be understood with the biomechanical framework that conceptualizes the neuroforamen as a dynamic, three-dimensional “box,” (Fig. 1D) whose volume and geometry are shaped by both segmental and global spinal alignment. Prior literature has shown that disc collapse, vertebral rotation, and alignment abnormalities—particularly on the concave side of a scoliotic curve—can cause a reduction in foraminal height by up to 50%, often decreasing it below the symptomatic threshold of 15 mm (Kelly et al., 2020; Kaneko et al., 2012). In our cohort, clinical outcomes following endoscopic decompression were strongly associated with radiographic indicators of such deformation. Specifically, patients with reduced lumbar lordosis experienced significantly worse postoperative back pain and less improvement in functional outcomes, reinforcing previous studies suggesting that hypolordosis decreases foraminal height by 3–4 mm due to anterior sagittal imbalance and posterior pelvic tilt. Similarly, those with larger Cobb angles—particularly those in the upper quartile (26–52°)—showed the least improvement in both leg and back pain and had higher ODI scores at follow-up. These findings align with existing data that every 10° increase in Cobb angle can decrease foraminal height on the concave side by 1.5–2 mm. The relationship between sagittal alignment and clinical response was further supported by outcome differences across SVA thresholds where patients with higher SVA exhibited diminished pain relief, likely due to compounded anterior column collapse and increased posterior element stress. These spinopelvic markers—particularly PI > 65° without adequate lordotic compensation—mirror the anatomical changes described in the literature, including posterior facet encroachment and narrowing of the posterior foraminal boundary. Our correlation analysis further reinforces the clinical relevance of these structural dynamics - CSVL deviation and Cobb angle were positively correlated with worsening leg pain, while higher PI was negatively associated with improvement in back pain. This multifactorial distortion—where sagittal and coronal malalignment compress the foramen in multiple directions—was reflected in our findings that patients meeting more favorable thresholds were more likely to achieve MCID for both VAS and ODI scores.

Structural instability further exacerbated these patterns. In our cohort, 36% of patients had lateral listhesis and 39% had spondylolisthesis—consistent with prior findings (Kleimeyer et al., 2019; Toyone et al., 2005). Both deformities likely contributed to foraminal narrowing via asymmetric loading, facet degeneration, and dynamic vertebral slippage (Pritchett and Bortel, 1993). Importantly, decompression performed within regions of maximal curvature, especially when complicated by listhesis or spondylolisthesis, was associated with higher rates of residual symptoms and mechanical instability—supporting prior biomechanical findings by Lafage et al. on altered load transmission in scoliotic spines (Lafage et al., 2009).

Physiologically, the similar outcomes from concave and convex decompression in our cohort reflect the complex three-dimensional mechanics of neural compression in ADS. Although the concave side is typically associated with foraminal narrowing, vertebral rotation and facet hypertrophy on the convex side can also cause nerve impingement (Kotwal et al., 2011). Asymmetric collapse may reduce foraminal space in all planes, not just on the concave side (Kotwal et al., 2011; Fu et al., 2011). These multidirectional forces likely explain why targeted endoscopic decompression yields symptom relief regardless of approach side. At L4–L5, high axial loading may predispose both sides to dynamic stenosis, minimizing differences between convex and concave interventions (Kim et al., 2013; Hiwatashi et al., 2004).

The reoperation rate in our cohort was 25%, with three patients undergoing subsequent fusion for persistent pain and the remainder benefitting from repeat endoscopic decompression. Specifically, two patients received decompression encompassing the original surgical level, while one received endoscopic decompression at the level above; all three of these patients received the same surgical approach for reoperation as the original procedure. Out of the three patients receiving subsequent fusion, all were inclusive of the original surgical level (L2-L4 PTP with posterior fusion; L4-S1 ALIF with L1-L4 PTP; and L2-L3 XLIF). The overall reoperation rate is slightly higher than previously reported figures of approximately 11%, potentially reflecting the longer follow-up period and the older average age of our patient population. However, it is also worth noting that endoscopic foraminotomy tends to have a higher reoperation rate than central canal decompression. The recent conception of the Extended Transforaminal Endoscopic Foraminotomy approach has been favored for better outcomes, cost-effectiveness, and lower reoperation rates compared to standard endoscopic decompression, and increased adoption of this technique may both improve outcomes and minimize complication rates moving forward (Ahn, 2025; Kim et al., 2025). Importantly, no other postoperative complications were observed. Patients who required reoperation exhibited significantly higher postoperative VAS scores for both back and leg pain, suggesting that persistent or recurrent symptoms following initial decompression may be predictive of the need for more extensive surgical intervention. Given the small size of the group requiring re-operation, we did not note any significant differences in spinopelvic parameters between those needing further surgery and those who did not.

### 4.1 Limitations

This study has several limitations. First, the small sample size and double-center design limit the generalizability of our findings. Patients with severe deformity were not captured, and our study did not control for certain confounding factors such as BMI, use of pain interventions, such as epidural steroid injections, facet blocks, or radiofrequency ablation, utility of post-operative physical therapy etc. Additionally, we did not perform co-group analysis to evaluate the interplay between multiple spinopelvic parameters (e.g., high PI and high SVA), which may have impacted clinical outcomes. Postoperative outcome measures, including VAS and ODI scores, were collected at variable time points, further introducing heterogeneity in follow-up data. Lastly, the follow-up duration of six months may be insufficient to capture long-term outcomes, particularly in patients with progressive deformity, as minimum one-to two-year follow-up is ideally preferred to capture recurrence or changes in Cobb's angle in ADS (Li et al., 2009). Future prospective studies with standardized protocols, longer follow-up, and multicenter collaboration will be necessary to validate and expand upon these findings.

In conclusion, our study offers new and clinically actionable insights into the anatomical predictors of endoscopic decompression success in ADS. By translating complex radiographic measurements into clear clinical thresholds, we provide a framework for risk stratification, treatment planning, and surgical decision-making. These findings move the field closer to evidence-based, patient-specific spine surgery, supporting the role of decompression in select patients while clarifying its limitations in the presence of significant deformity or instability. As MIS techniques continue to evolve, such alignment-informed protocols will be essential to improving outcomes and minimizing the need for reoperation in this challenging patient population.

## 5 Conclusions

This multi-institutional study supports transforaminal endoscopic decompression as an effective treatment for symptom relief in select adult degenerative scoliosis patients, particularly those with primary leg pain and mild to moderate deformity. We identified key spinopelvic parameters—such as Cobb angle, lumbar lordosis, and PI —that correlate with improved postoperative pain and functional outcomes. While endoscopic decompression may not be suitable for all ADS patients, particularly those with severe coronal or sagittal imbalance or significant segmental instability, it offers a valuable alternative to open fusion procedures in moderate deformity cases, especially among older patients with higher surgical risk. Future prospective, multicenter studies with larger cohorts and long-term follow-up are needed to validate these findings and refine selection criteria. Nonetheless, our study represents an important step toward individualized, pre-operative parameter-based treatment strategies for patients with radiculopathy secondary to ADS.

## Disclosures

The authors have no disclosures relevant to the current work, nor any true/perceived conflicts of interest.

## Funding

This research did not receive any specific grant from funding agencies in the public, commercial, or not-for-profit sectors.

## Declaration of interests

The authors declare that they have no known competing financial interests or personal relationships that could have appeared to influence the work reported in this paper.
