# Supplementary material for: Radiographic and clinical predictors of surgical outcomes following endoscopic decompression for radiculopathy in adult degenerative scoliosis: A multi-center retrospective study
Source: Brain Spine. 2026 Mar 3;6:105990. doi: 10.1016/j.bas.2026.105990 (PMC12993231; doi:10.1016/j.bas.2026.105990)
Supplement: Multimedia component 1 [file mmc1.docx]

**Radiographic and Clinical Predictors of Surgical Outcomes Following Endoscopic Decompression for Radiculopathy in Adult Degenerative Scoliosis: A Multi-center Retrospective Study**

SUPPLEMENTAL MATERIALS

**Table S1.** Pain outcomes by symptom type

| Symptom Type | Improved (%) | Worsened (%) | Unchanged (%) |
| --- | --- | --- | --- |
| Leg pain | 75 | 21 | 3.5 |
| Back pain | 61 | 32 | 7 |

**Table S2.** Type and Laterality of Stenosis (n = 18)

|  | **Number (n)** | **Percent (%)** |
| --- | --- | --- |
| Type of Stenosis** |  |  |
| Foraminal | 18 | 52.9% |
| Lateral | 15 | 44.1% |
| Transforaminal | 1 | 2.9% |
| Laterality of Stenosis |  |  |
| Bilateral | 8 | 44.4% |
| L | 8 | 44.4% |
| R | 2 | 11.1% |

*Only single-institution data available for n = 18, included in this table

**Type of Stenosis can include multiple types, so does not total n = 18

**Table S3**. Surgical characteristics for patients receiving neurosurgical reoperation

| Deidentified patient number | Level(s) and approach for original surgery | Time to reoperation | Reoperation indication | Level(s) and approach for reoperation |
| --- | --- | --- | --- | --- |
| 1 | L L3/L4 TF discectomy with decompression | 159 days | L2-L4 spondylolisthesis | L2-L4 PTP with posterior fusion |
| 2 | R L4/L5 TF discectomy with decompression | 561 days | L4/L5 stenosis with radiculopathy | L4-S1 decompression |
| 3 | R L4/L5 TF discectomy with decompression | 97 days | Residual nerve compression | Focal lower extremity decompression |
| 4 | L L4/L5 TF discectomy with decompression | 238 days | L5/S1 spondylolisthesis | L4-S1 ALIF with L1-L4 PTP |
| 5 | TELD | 0 days | Pain and radiculopathy in lower extremity | L2/L3 XLIF |
| 6 | TELF | 663 days | Pain and radiculopathy in lower extremity | L4/L5 TELF |
| 7 | TELD | 191 days | Pain and radiculopathy in lower extremity | L2/L3 TELD |

**Figure S1**. ODI scores at last post-operative visit by re-operation status * demonstrates statistical significance at p < 0.05.

**
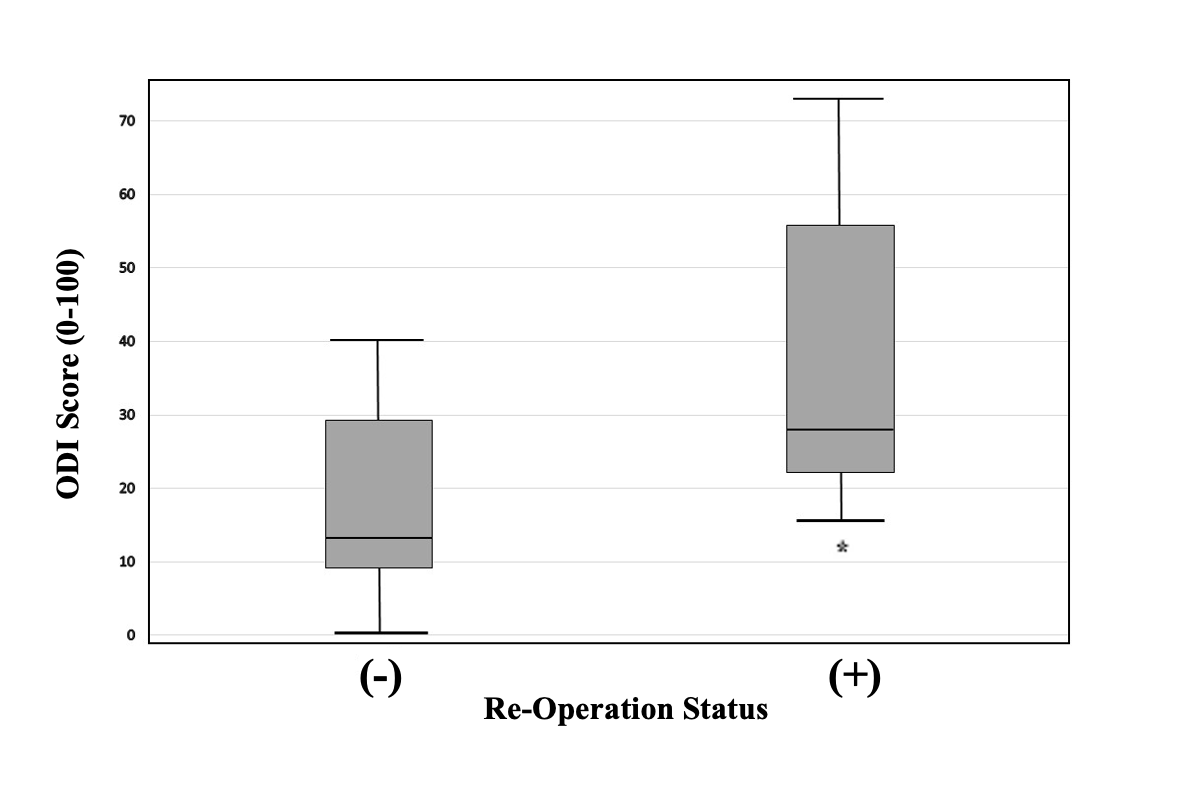
**

**Figure S2.** ODI scores at last post-operative correlated with presence of a) lateral listhesis or b) spondylolisthesis at the level of surgery on pre-operative imaging * demonstrates statistical significance at p < 0.05.

**
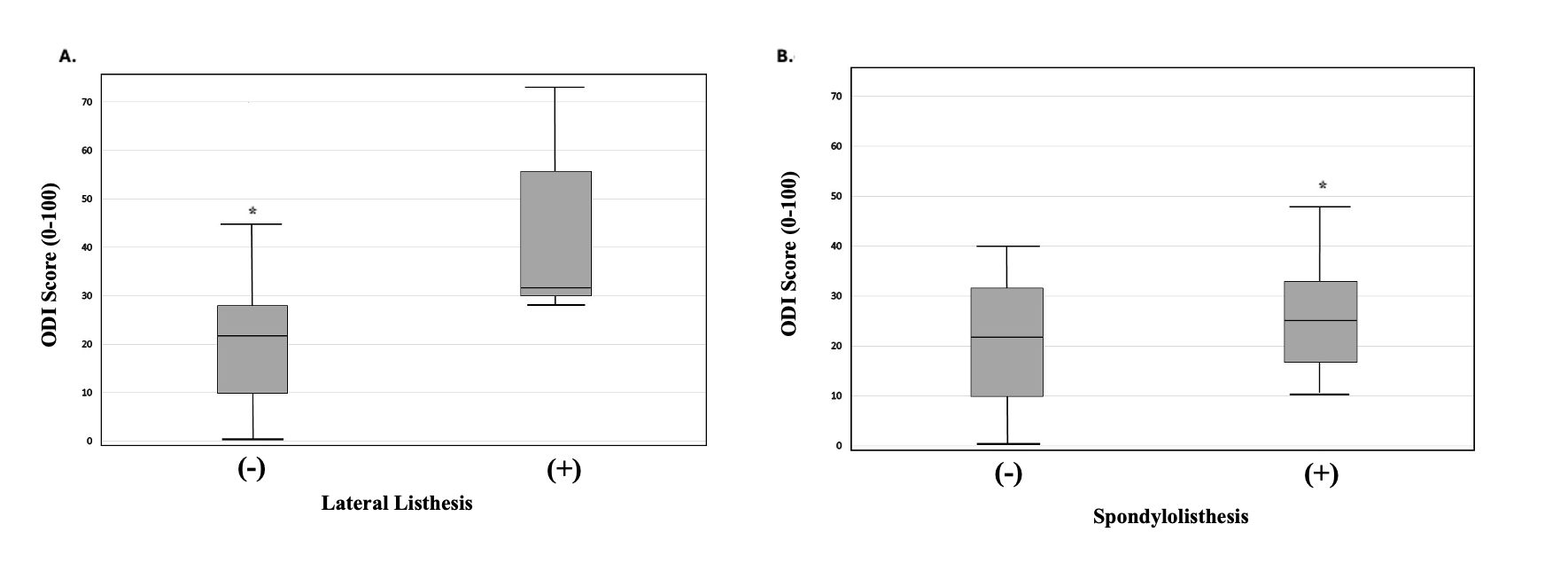
**
